# Supplementary material for: Immunotherapy and Cannabis: A Harmful Drug Interaction or Reefer Madness?
Source: Cancers (Basel). 2024 Mar 22;16(7):1245. doi: 10.3390/cancers16071245 (PMC11011043; doi:10.3390/cancers16071245)
Supplement: Supplementary file 1 [file cancers-16-01245-s001.zip › cancers-2868537-File S2.pdf]

Table S1. Examples of studies that have cited the Teha [8] and the Bar-Sela [9] cannabis and immunotherapy manuscripts.

Authors	Journal	Title	Conclusions
Bodine and Kemp [1]	*StatPearls*	Medical Cannabis Use in Oncology	″when utilizing medicinal marijuana to treat the side effects associated with oncology treatment regimens, clinicians must consider the possibility of cannabinoids interfering with the effectiveness of their patient's cancer therapy″
To et al. [2]	*Supportive Care in Cancer*	MASCC Guideline: cannabis for cancer-related pain and risk of harms and adverse events	"We recommend against using cannabinoids for any indication in cancer patients undergoing treatment with a checkpoint inhibitor (level of evidence: III; grading of evidence: C; category of guideline: suggestion)"
Ramer et al. [3]	*Cancers*	Impact of Cannabinoid Compounds on Skin Cancer	"It seems likely that the obvious combination of cannabinoids and checkpoint inhibitors is a rather unfavorable variant of combination therapy"
Abrams et al. [4]	*JNCI Monographs*	Cancer Treatment: Preclinical & Clinical	"clinicians should now apprise patients embarking on immunotherapy regimens of these findings so they may be aware of the potential risks"
Nugent et al. [5]	*Cancer*	Medical Cannabis Use Among Individuals with Cancer: An Unresolved and Timely Issue	"The coadministration of cannabinoids and chemotherapeutics with the potential for drug-drug interactions via any of these pathways is discouraged"
Creanga-Murariu et al. [6]	*Frontiers in Pharmacology*	Should Oncologists Trust Cannabinoids?	"the scarce clinical data available more likely indicate a deleterious effect of cannabinoids on tumors exposed to immunotherapy"
Nahler [7]	*Pharmaceutical Medicine*	Cannabidiol and Other Phytocannabinoids as Cancer Therapeutics	"Caution is also advised against the use of cannabis by cancer patients during immune therapy with monoclonal antibodies, as this may result in a decrease in time to tumor progression and decreased overall survival"
Abu-Amna et al. [8]	*Current Treatment Options in Oncology*	Medical Cannabis in Oncology: A Valuable Unappreciated Remedy or an Undesirable Risk?	"In conclusion, we recommend using cannabis with caution in oncology patients being treated with immunotherapy and suggest prescribing cannabis only when there are clear indications and expected benefits"

Sarsembayeva et al. [9]	*Frontiers in Oncology*	Cannabinoids and endocannabinoids system in immunotherapy: helpful or harmful?	"Likewise, the use of medical cannabis or cannabis-derived products during immunotherapy should be reconsidered as they might interfere with ICIs` mechanism"
Hinz et al. [10]	*British Journal of Cancer*	Cannabinoids as anticancer drugs: status of preclinical research	"This study illustrates that cannabis use, in the case via modulation of the immune system, can lead to negative and thus life-threatening effects for cancer patients."

Citations

1. Bodine, M.; Kemp, A.K. Medical Cannabis Use in Oncology. In *StatPearls*; StatPearls Publishing: Treasure Island (FL), 2024.
2. To, J.; Davis, M.; Sbrana, A.; Alderman, B.; Hui, D.; Mukhopadhyay, S.; Bouleuc, C.; Case, A.A.; Amano, K.; Crawford, G.B.; et al. MASCC Guideline: Cannabis for Cancer-Related Pain and Risk of Harms and Adverse Events. *Support Care Cancer* **2023**, *31*, 202, doi:10.1007/s00520-023-07662-1.
3. Ramer, R.; Wendt, F.; Wittig, F.; Schäfer, M.; Boeckmann, L.; Emmert, S.; Hinz, B. Impact of Cannabinoid Compounds on Skin Cancer. *Cancers (Basel)* **2022**, *14*, 1769, doi:10.3390/cancers14071769.
4. Abrams, D.I.; Velasco, G.; Twelves, C.; Ganju, R.K.; Bar-Sela, G. Cancer Treatment: Preclinical & Clinical. *J Natl Cancer Inst Monogr* **2021**, *2021*, 107–113, doi:10.1093/jncimonographs/lgab010.
5. Nugent, S.M.; Meghani, S.H.; Rogal, S.S.; Merlin, J.S. Medical Cannabis Use among Individuals with Cancer: An Unresolved and Timely Issue. *Cancer* **2020**, *126*, 1832–1836, doi:10.1002/cncr.32732.
6. Creanga-Murariu, I.; Filipiuc, L.E.; Cuciureanu, M.; Tamba, B.-I.; Alexa-Stratulat, T. Should Oncologists Trust Cannabinoids? *Frontiers in Pharmacology* **2023**, *14*, doi:10.3389/fphar.2023.1211506.
7. Nahler, G. Cannabidiol and Other Phytocannabinoids as Cancer Therapeutics. *Pharmaceut Med* **2022**, *36*, 99–129, doi:10.1007/s40290-022-00420-4.
8. Abu-Amna, M.; Salti, T.; Khoury, M.; Cohen, I.; Bar-Sela, G. Medical Cannabis in Oncology: A Valuable Unappreciated Remedy or an Undesirable Risk? *Curr. Treat. Options in Oncol.* **2021**, *22*, 16, doi:10.1007/s11864-020-00811-2.
9. Sarsembayeva, A.; Schicho, R. Cannabinoids and the Endocannabinoid System in Immunotherapy: Helpful or Harmful? *Front Oncol* **2023**, *13*, 1296906, doi:10.3389/fonc.2023.1296906.
10. Hinz, B.; Ramer, R. Cannabinoids as Anticancer Drugs: Current Status of Preclinical Research. *Br J Cancer* **2022**, *127*, 1–13, doi:10.1038/s41416-022-01727-4.

Table S2. Percentages reported from [9] and calculated for Cannabis Users (CU) and Cannabis Non Users (CNU). ECOG: Eastern Conference Oncology Group.

Variable	Numerator	Denominator	Reported %	Calculated %	Difference
Gender-Female (CU)	10	34	29.5%	29.4%	-0.1%
Gender-Male (CU)	24	34	70.5%	70.6%	+0.1%
ECOG $\leq$ 1 (CNU)	55	68	80.8%	80.9%	+0.1%
ECOG $\leq$ 1 (CU)	24	34	70.5%	70.6%	+0.1%
Chronic diseases = 0 (CNU)	22	68	32.3%	32.4%	+0.1%
Chronic diseases = 0 (CU)	13	34	22.0%	38.2%	+16.2%
Chronic diseases = 1 (CU)	7	34	20.5%	20.6%	+0.1%
Chronic diseases = 2 (CU)	14	34	41.1%	41.2%	+0.1%
Chronic heart disease (CNU)	18	68	26.4%	26.5%	+0.1%
High blood pressure (CU)	13	34	34.1%	38.2%	+4.1%
Melanoma (CNU)	25	68	36.7%	36.8%	+0.1%
Melanoma (CU)	9	34	26.4%	26.5%	+0.1%
Renal cell carcinoma (CNU)	4	68	5.8%	5.9%	+0.1%
Renal cell carcinoma (CU)	2	34	5.8%	5.9%	+0.1%
Brain metastasis (CU)	8	34	13.2%	23.5%	+10.3%
Lungs metastasis (CNU)	39	68	57.3%	57.4%	+0.1%
Liver metastasis (CU)	11	34	32.3%	32.4%	+0.1%
Immunotherapy 1st line (CNU)	31	68	45.5%	45.6%	+0.1%
Immunotherapy 2nd line (CU)	26	34	76.4%	76.5%	+0.1%
Pembrolizumab of nivolumab (CU)	29	34	85.2%	85.3%	+0.1%
Ipilimumab or nivolumab (CU)	4	34	11.7%	11.8%	+0.1%
Durvalumab or atezolizumab (CNU)	5	68	7.3%	7.4%	+0.1%

Supplementary Text S1. British Medical Journal guidance [20] on the selection of non-parametric statistics for small N.

"When the numbers in a 2 x 2 contingency table are small, the χ^2 approximation becomes poor. The following recommendations may be regarded as a sound guide. In fourfold tables a χ^2 test is inappropriate if the total of the table is less than 20, or if the total lies between 20 and 40 and the smallest expected (not observed) value is less than 5; in contingency tables with more than one degree of freedom it is inappropriate if more than about one fifth of the cells have expected values less than 5 or any cell an expected value of less than 1. An alternative to the χ^2 test for fourfold tables is known as Fisher's Exact test and is described in Chapter 9

When the values in a fourfold table are fairly small a "correction for continuity" known as the "Yates' correction" may be applied. Although there is no precise rule defining the circumstances in which to use Yates' correction, a common practice is to incorporate it into χ^2 calculations on tables with a total of under 100 or with any cell containing a value less than 10."
